# Prehospital Medication Administration: A Randomised Study Comparing Intranasal and Intravenous Routes

**DOI:** 10.1155/2012/476161

**Published:** 2012-08-16

**Authors:** Cian McDermott, Niamh C. Collins

**Affiliations:** ^1^Centre for Emergency Medical Science, University College Dublin, Dublin, Ireland; ^2^Medical Advisory Group of the Pre-hospital Emergency Care Council in Ireland, Naas, Ireland

## Abstract

*Introduction*. Opioid overdose is an ever-increasing problem globally. Recent studies have demonstrated that intranasal (IN) naloxone is a safe and effective alternative to traditional routes of naloxone administration for reversal of opioid overdose. *Aims*. This randomised controlled trial aimed to compare the time taken to deliver intranasal medication with that of intravenous (IV) medication by advanced paramedic trainees. *Methods*. 18 advanced paramedic trainees administered either an IN or IV medication to a mannequin model in a classroom-based setting. The time taken for medication delivery was compared. End-user satisfaction was assessed using a 5-point questionnaire regarding ease of use and safety for both routes. *Results*. The mean time taken for the IN and IV group was 87.1 seconds and 178.2 seconds respectively. The difference in mean time taken was 91.1 seconds (95% confidence interval 55.2 seconds to 126.9 seconds, *P* ≤ 0.0001). 89% of advanced paramedic trainees reported that the IN route was easier and safer to use than the IV route. *Conclusion*. This study demonstrates that, amongst advanced paramedic trainees, the IN route of medication administration is significantly faster, better accepted and perceived to be safer than using the IV route. Thus, IN medication administration could be considered more frequently when administering emergency medications in a pre-hospital setting.

## 1. Introduction

The mortality associated with opioid overdose has continued to increase globally in recent years. In 2009, the number of Irish drug-related deaths attributed to opioid intoxication rose by 20% [1], while in Europe, opioids were responsible for 75% of all drug-related deaths [2]. In the United States in 2007, there were 11,499 deaths resulting from opioid overdose [3]. The main cause of death is as a result of opioid-induced respiratory depression [4]. After the initiation of basic life support measures, naloxone is an opioid antagonist that is used to reverse respiratory depression and mental state changes. It is widely marketed under the brand name Narcan. The common routes of administration of naloxone are intravenous (IV), intraosseous (IO), intramuscular (IM), and subcutaneous. Intranasal (IN) administration is an alternative route for naloxone delivery [5].

When a patient presents in opioid-induced cardiorespiratory arrest, immediate effective antagonism by naloxone reverses the opioid-induced side effects. Direct entry of naloxone into the systemic circulation is required and this is most reliably achieved with IV or IO medication administration. Vascular access is often a major challenge when treating a patient with opioid overdose in the prehospital setting due to damage to veins from repeated drug use [6]. Multiple attempts at intravenous cannulation may result in an increased risk of exposure to blood-borne infections, in a group of patients that have a high seroprevalence of blood-borne transmissible viral infections (hepatitis B, C, and human immunodeficiency virus) [6]. The rate of occupational blood exposures for prehospital providers is estimated to be in excess of 49,000 per annum, which includes over 10,000 cases of needlestick injuries [7]. 

Most opioid overdoses occur in a prehospital setting, arising from unintentional self-poisoning [8]. Emergency medical services (EMS) providers are usually the patient's first contact with the health service. In many jurisdictions worldwide, naloxone is used by EMS personnel to treat opioid overdoses [9–13]. In Ireland, the prehospital emergency medical care system is regulated and governed by the Pre-Hospital Emergency Care Council [14]. EMS personnel (paramedics and advanced paramedics) are permitted to administer naloxone to treat a suspected opioid overdose in accordance with national clinical practice guidelines [15]. However, there is currently no provision for the use of IN naloxone in prehospital medicine in Ireland. The introduction of an alternative needle-free route of naloxone delivery that is fast acting, effective, and safe would be beneficial to patients and EMS providers.

Intranasal administration of naloxone obviates the need for IV catheter placement in high-risk patients and could reduce some of these associated risks. The nasal route is presented as an alternative for drug delivery since the rich vascular plexus of the nose offers a direct route for medication entry into the bloodstream [5, 8]. Also, especially relevant to prehospital clinical practice, the nasal cavity is a readily accessible and pain-free site for use in any emergency situation.

While the bioavailability of IN naloxone reaches almost 100% that of IV naloxone and achieves peak plasma concentration in 3 minutes in animal studies [16], there is a lack of human pharmacokinetic data. Previous studies have demonstrated that IN naloxone is effective and safe when used to treat an opioid overdose [9–11]. Several non-randomised pre-hospital studies have also shown that the overall time interval from patient contact to patient recovery is similar for IN and IV naloxone [12, 13]. 

The primary aim of this study is to compare the time taken to administer a medication via the IN and IV routes. A secondary aim is to assess the end-user satisfaction for both routes in a cohort of advanced paramedic trainees. 

## 2. Methods

### 2.1. Study Setting and Design

**Figure 1:**
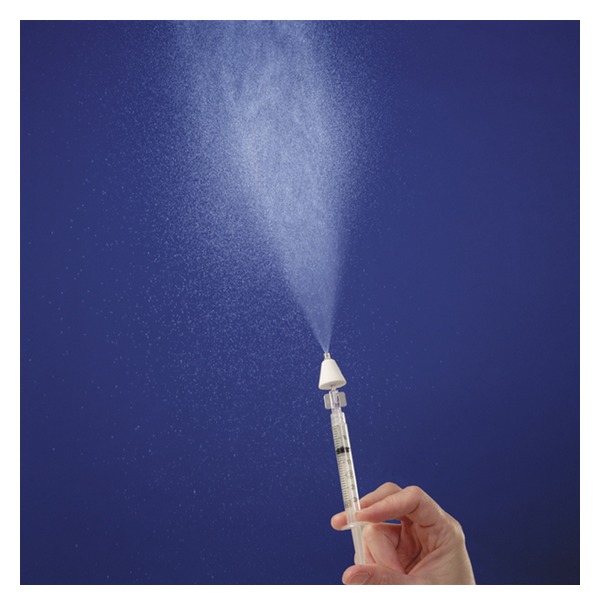
Mucosal atomizer device for delivery of intranasal medication (reproduced with permission from Wolfe Tory Medical, Inc., USA).

This was a randomised controlled trial that took place at the National Ambulance Services College in Dublin, Ireland. A class of 18 advanced paramedic trainees, registered with a University College Dublin training programme, were asked to participate in a classroom-based study that was used to simulate a real-life patient encounter of an opioid overdose. Standardised formal IV cannulation techniques had previously been taught using a mannequin and each trainee had completed a five-week hospital placement during which time supervised IV cannulations were performed on patients. Each trainee also received formal instruction regarding the use of a mucosal atomizer device (MAD) to deliver intranasal medication. This is a single-use atomizer device with a luer-lock connector for delivery of a measured dose of IN medication via a syringe (Figure 1).

Block randomisation was used to assign trainees equally to each study group—9 trainees were allocated to group A (IN) and the remainder was assigned to group B (IV). 

**Figure 2:**
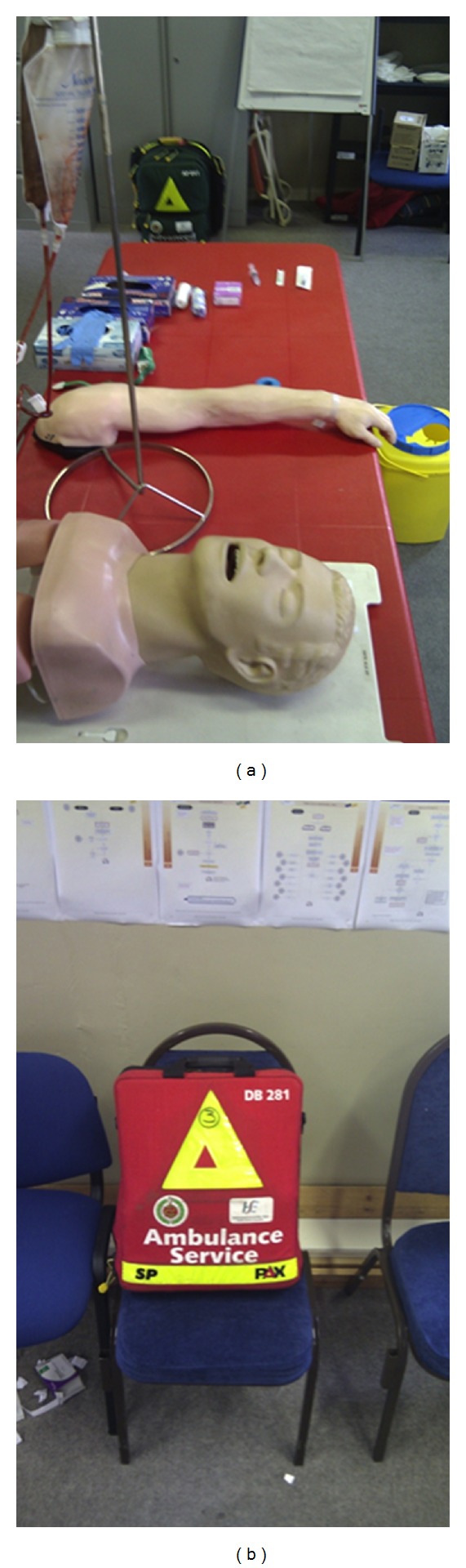
Materials used to carry out classroom-based study in the National Ambulance Services Centre.

The study was designed to mirror a real-life patient encounter. A table was arranged at bed height with a mannequin for IN administration and a phlebotomy arm for IV cannulation (Figure 2). A standard advanced paramedic kit bag, containing the MAD, a 3 ml plastic syringe, a 21G hypodermic needle, and a 20G IV cannula in a clear plastic pouch was placed beside the table. A clear glass vial, filled with 1 ml of saline solution was used for both groups. Trainees were instructed to administer the medication as per the route indicated at randomisation. A research assistant who was not involved in the study design or result interpretation recorded the time taken for each trainee to prepare the medication and prepare the route of administration (i.e., insert a cannula or check the nose). The clock was started as the trainee opened the kit bag and stopped as the medication was delivered. Each trainee was permitted to complete the task once only.

### 2.2. Outcome Measures

The primary outcome measure in this study was the time taken by trainees for completion of the task in group A (IN) and group B (IV) as detailed above. 

Practitioner satisfaction with each route of medication administration was the secondary outcome measure. Following completion of the procedure, each trainee was asked to fill out a 5-point Likert rating scale. This was used to measure the trainees' satisfaction in terms of user-friendliness and safety of the procedure that they had been assigned to. A procedure was defined as “safe” if the trainee did not expect to encounter a blood exposures or needlestick injury while using that technique in a real-life scenario. 

### 2.3. Data Analysis

Descriptive statistical analysis was applied to the data in this study (mean, median, standard deviation and mean time difference with 95% confidence intervals, CI). The data was found to follow a normal distribution using the Anderson-Darling test; thus, the difference in mean times for both groups was compared using a two-tailed student's *t*-test. A *P*-value < 0.05 was chosen as significant. 

## 3. Results

18 advanced paramedic trainees participated in this study—15 males and 3 females. The mean age of participants was 50.5 years and the age range was 32 years to 57 years.  Table 1 compares the route of medication administration and time taken for each advanced paramedic trainee.

The mean time taken for group A to deliver medication via the IN route was 87.1 seconds. The standard deviation was 20.35 (range 57.4 to 114.9 seconds). The mean time taken for group B to insert a cannula and administer the medication IV was 178.2 seconds. The standard deviation was 36.71 (range 133.7 to 240.6 seconds). There was a difference in mean delivery times of 91.1 seconds (*P* ≤ 0.0001) with 95% CI ranging from 55.2 seconds to 126.9 seconds. Thus, there was a statistically significant difference in the primary outcome measure in this study in favour of IN medication administration. 

**Figure 3:**
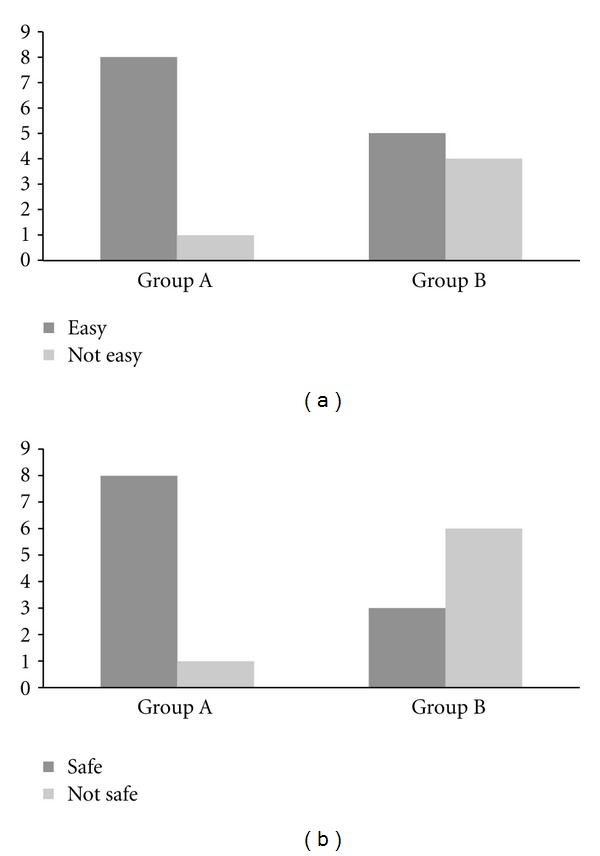
(a) Advanced paramedic trainee response to questionnaire regarding ease of use of IN or IV delivery. (b) Advanced paramedic trainee response to questionnaire regarding safety of use of IN or IV delivery.

Eighty-nine percent (8 out of 9) of trainees from group A “strongly agreed” that the IN technique was both easy to use and safe to use. Most trainees from group B regarded the IV technique as easy to use but most “disagreed” (67%) that the technique was safe to use (Figures 3(a) and 3(b)). All trainees completed the study and no adverse incidents occurred.

## 4. Discussion

The findings of this study show that it is faster to deliver a medication via the IN route than the IV route when administered by a cohort of advanced paramedic trainees. To our knowledge, no study has yet attempted to quantify the actual time difference that occurs as a result of the route of administration used to deliver naloxone. In this study, the IN route was also preferred over the IV route, both in terms of ease of use and safety profile. 

Two randomised controlled trials have compared the time taken to achieve adequate patient response when using IN and IM naloxone [9, 10]. A positive clinical response in both of these studies was defined as the time taken to regain a respiratory rate of 10 breaths per minute. Patients in the initial study had a slower response when given IN naloxone (IN 8 minutes versus IM 6 minutes, *P* = 0.006) [10] while mean response times were similar in the more recent study (IN 8.0 minutes, IM 7.9 minutes, difference 0.1, 95% CI −1.3 to 1.5) [9]. A more concentrated solution of IN naloxone was specifically manufactured for use in the later study—this was thought to account for the difference in response time for IN naloxone between these studies.

Additional nonrandomised studies have shown that the overall time intervals from initial patient contact by paramedics to patient clinical response (defined as an increase in respiratory rate and Glasgow Coma Score) were not prolonged when using IN naloxone compared with IV naloxone [12, 13]. The authors concluded that any delay in the clinical response to IN naloxone is compensated for by the time taken to establish IV access.

A mean time difference of 91.1 seconds was recorded in this study with the 95% confidence interval ranging from 55.2 seconds to 126.9 seconds. A clinically significant difference in patient response times has previously been defined as 1 minute, based on respiratory depression and oxygen desaturation that may occur after this time [9]. Thus, the use of the IN route of delivery of naloxone to treat an opioid overdose may have an important impact on successful patient resuscitation in a real-life clinical scenario.

The results of this study also concluded that there was high level of practitioner satisfaction among advanced paramedic-trainees in relation to the ease of use of the IN route of administration. In this cohort, 89% of users found the IN route easy to use. Paramedics in other studies perceived IN naloxone to be less effective than its parenteral counterpart [11]. It has been reported that there is a preference by paramedics toward one route of delivery or another based on personal experience and not on the level of patient intoxication [11]. However, advanced paramedic trainees in this study expressed a clear preference for the IN route.

In the United States (US), in 2000, the Needlestick Safety and Prevention Act was enacted into federal law [17]. Under this new legislation, the Occupational Safety and Health Administration established requirements for all employers to reduce percutaneous injuries in at-risk employees from contaminated sharps by using safety-engineered medical devices [18]. Prior to this, the rate of needlestick injury was estimated at 378,000 to 756,000 incidents per annum [19]. Since its introduction, there has been a steady decline in the annual rate of percutaneous injuries in the US, for example, in 2001, a reduction of almost 38% was reported amongst hospital employees [20]. The results of this study show that most advanced paramedic trainees perceived the IN route (89%) to be safer than the IV route of administration (33%). 

Thus, IN naloxone is proposed as one such needle-free initiative that may reduce exposure of EMS personnel to blood-borne viruses, when treating high-risk patients with an opioid overdose. 

## 5. Limitations

The limitations of this study include its small sample size (*n* = 18) and that it lacked blinding. The small sample size was due to the availability of advanced paramedic trainees that were enrolled in the teaching programme at the time of the study. Also, the participants were advanced paramedic trainees and may not yet have sufficient experience in IV cannulation techniques, which may have increased the time taken to gain IV access in some cases. Finally, this was a classroom-based study designed to simulate real-life events. In clinical practice, a field-based patient encounter may have other confounding patient and environmental variables that could potentially affect the outcomes. 

## 6. Conclusion

This study demonstrates that, amongst advanced paramedic-trainees, the IN route of medication administration is significantly faster, better accepted, and perceived to be safer than using an IV route of administration. The authors therefore, propose that this needle-free route of medication administration be employed more frequently when treating high-risk patients with an opioid overdose. 

## Figures and Tables

**Table 1:** Advanced paramedic trainees shown by time taken for medication delivery.

Trainee	Group A IN (s)	Group B IV (s)
1		185.4
2		159.4
3		240.6
4	103.8	
5	103.4	
6		133.7
7		231.6
8		152.2
9	95.7	
10	82.3	
11	114.9	
12		186.2
13	95.3	
14	68.8	
15	62.3	
16		161
17		153.4
18	57.4	
